# Anti-hyperglycemic and anti-hyperlipidemia effects of the alkaloid-rich extract from barks of *Litsea glutinosa* in ob/ob mice

**DOI:** 10.1038/s41598-018-30823-w

**Published:** 2018-08-23

**Authors:** Xiaopo Zhang, Yan Jin, Younan Wu, Caiyun Zhang, Dejun Jin, Qingxia Zheng, Youbin Li

**Affiliations:** 1 School of Pharmaceutical Science, Hainan Medical University, Haikou, 571199 China; 2 Zhengzhou Tobacco Research Institute, Zhengzhou, 450001 China

## Abstract

The present study investigated the anti-hyperglycemic and anti-hyperlipidemia effects of the alkaloid-rich extract from *Litsea glutinosa* barks (CG) in ob/ob mice. CG was orally administrated (50, 100 and 200 mg/kg) to ob/ob mice for 4 weeks. Parameters of glucose metabolism, hepatotoxicity, hyperlipidemia and inflammation were measured. CG was chemically characterized using UPLC-QTOF-MS. CG dose-dependently decreased body and fat weights without reducing average food intake. CG (100–200 mg/kg) significantly reduced the serum levels of fasting glucose, glycosylated hemoglobin (HbAlc) and glycosylated serum protein (GSP). CG increased insulin sensitivity as manifested by decreased fasting serum insulin, reduced homeostasis model assessment-estimated insulin resistance (HOMA-IR) and improved oral glucose tolerance. CG also alleviated dyslipidemia, ameliorated liver steatosis, increased the activity of serum lipase and alleviated inflammation. The activities of liver pyruvate kinase and glucokinase as well as liver content of glycogen were increased after CG treatment. CG was rich in alkaloids and eight main alkaloids were identified, many of which had been demonstrated to possess adequate anti-diabetic activities. These results suggest that the alkaloid-rich extract of CG possesses potential anti-hyperglycemic and anti-hyperlipidemic effects and can be utilized as an effective agent for the treatment of type 2 diabetes.

## Introduction

Type 2 diabetes (T2DM) is a global epidemic which is usually associated with hyperglycemia, hyperinsulinemia, and hyperlipidemia^1,2^. Insulin resistance (IR) is the typical character and precedes the development of T2DM^3^. Deficiency in insulin signal transduction, glycogen synthesis and over-accumulation of triglycerides have been implicated as key factors of IR^4,5^. Although the currently used anti-diabetic medications are adequate for the control of blood glucose, new effective anti-diabetic drugs with multiple benefits on T2DM associated syndromes are still tremendously needed to deal with this systemic metabolic disease.

*Litsea glutinosa* belongs to the family Lauraceae and is a well-known aromatic tree known in China as “Chan Gao Shu”. The leaves and barks of *L. glutinosa* are traditionally used as a demulcent and mild astringent for diarrhea and dysentery, whereas the paste of its root is used by the local residents to treat poultice sprains and bruises^6^. Phytochemical studies have obtained alkaloids, lignans, and flavones from the leaves and barks of this plant^6–9^. Recently, we have isolated three new lignans from the root barks of *L. glutinosa*^10^. The leaves and barks of *L. glutinosa* have reported to possess antibacterial, anti-inflammatory, anti-nociceptive, analgesic, and anti-diabetic properties^11,12^.

In the present study, the anti-hyperglycemic and anti-hyperlipidemia effects of CG were evaluated in ob/ob diabetic mice. The effect of CG on serum glucose and lipid profiles, insulin sensitivity, and hepatic glucose metabolism were assessed. The potential mechanisms of the anti-hyperglycemic effect of CG were also explored.

## Results

### CG was rich in alkaloids

**Figure 1:**
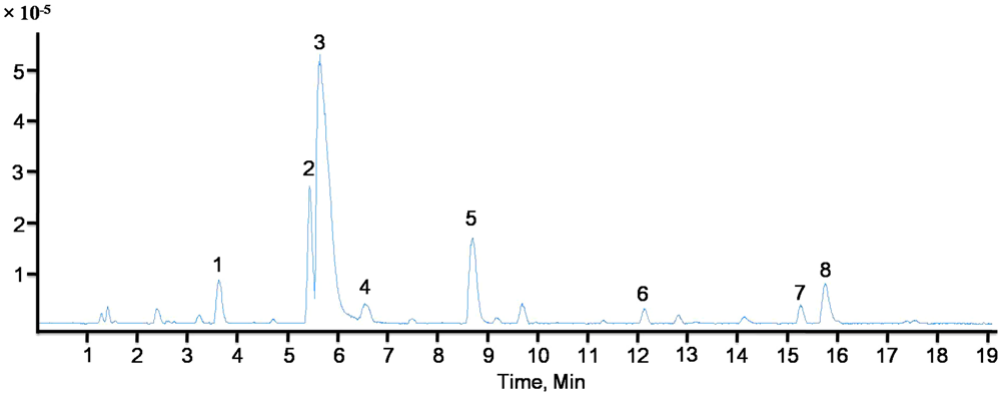
UPLC-QTOF-MS spectrum of CG (total ion).

UPLC-QTOF-MS analysis revealed that the CG was rich in alkaloids. Approximately thirteen visible peaks could be determined from the total ion current profile of the CG, of which eight were identified as alkaloids, including five aporphine and three benzylisoquinoline alkaloids. The total ion chromatography of CG was depicted in Figs 1 and S1, respectively. The compounds were characterized by their retention times and their mass spectral data and were identified by comparison with published data. They were identified as Laurelliptine (1), 6-Isoquinolinol (2), Laurolitsine (3), Isoboldine (4), N-methyl laurolitsine (5), Laurolitsine (6), Boldine (7), Litseglutine A (8). Their chemical structures were showed in Fig. S1.

### CG reduced obesity without restricting food intake

**Figure 2:**
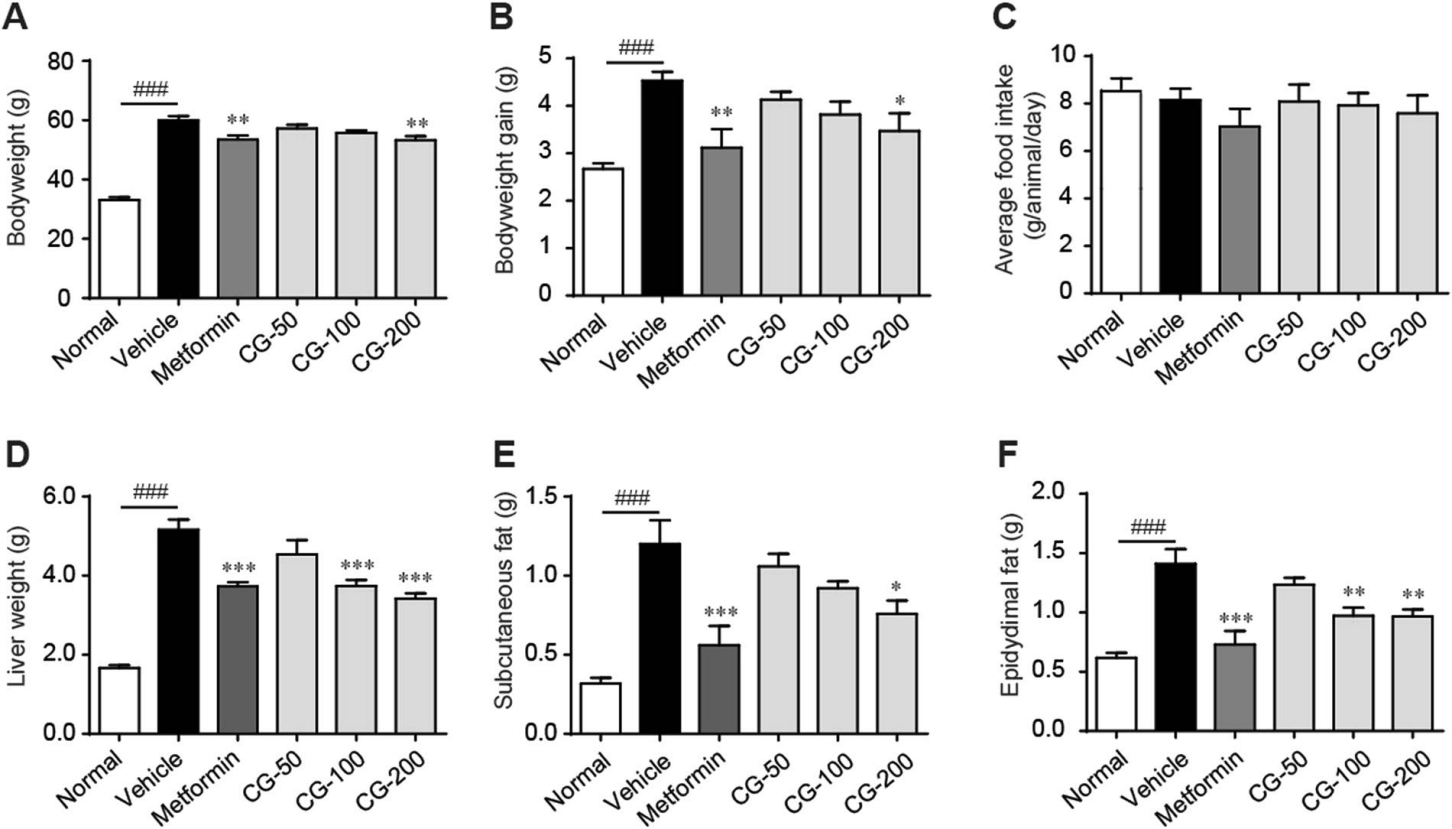
CG decreased body weight in ob/ob mice. (**A**) Body weight. (**B**) Body weight gain during the 4-week period. (**C**) Average food intake. (**D**) Liver weight. (**E**) Subcutaneous fat weight. (**F**) Epidy-dimal fat weight. Six animals in each group. ^#^*p* < 0.05, ^###^*p* < 0.001, normal group vs vehicle control group; *p < 0.05, **p < 0.01, ***p < 0.001, medication groups vs vehicle control group.

As compared to the lean control, the ob/ob mice showed obvious obesity as indicated by the significant increase in body weight and liver and fat weights (Fig. 2A–F). The body weight and body weight gain were dose-dependently reduced after treating with CG for 4 weeks (Fig. 2A and B). The increases in liver and fat (subcutaneous fat and epidy-dimal fat) weights were significantly decreased by CG at the doses of 200 mg/kg (Fig. 2D–F). The efficiency of CG in preventing obesity was comparable to metformin. Interestingly, metformin significantly reduced the average food intake while CG showed little influence on food intake (Fig. 2C).

### CG decreased blood glucose and serum HbAlc and GSP

**Figure 3:**
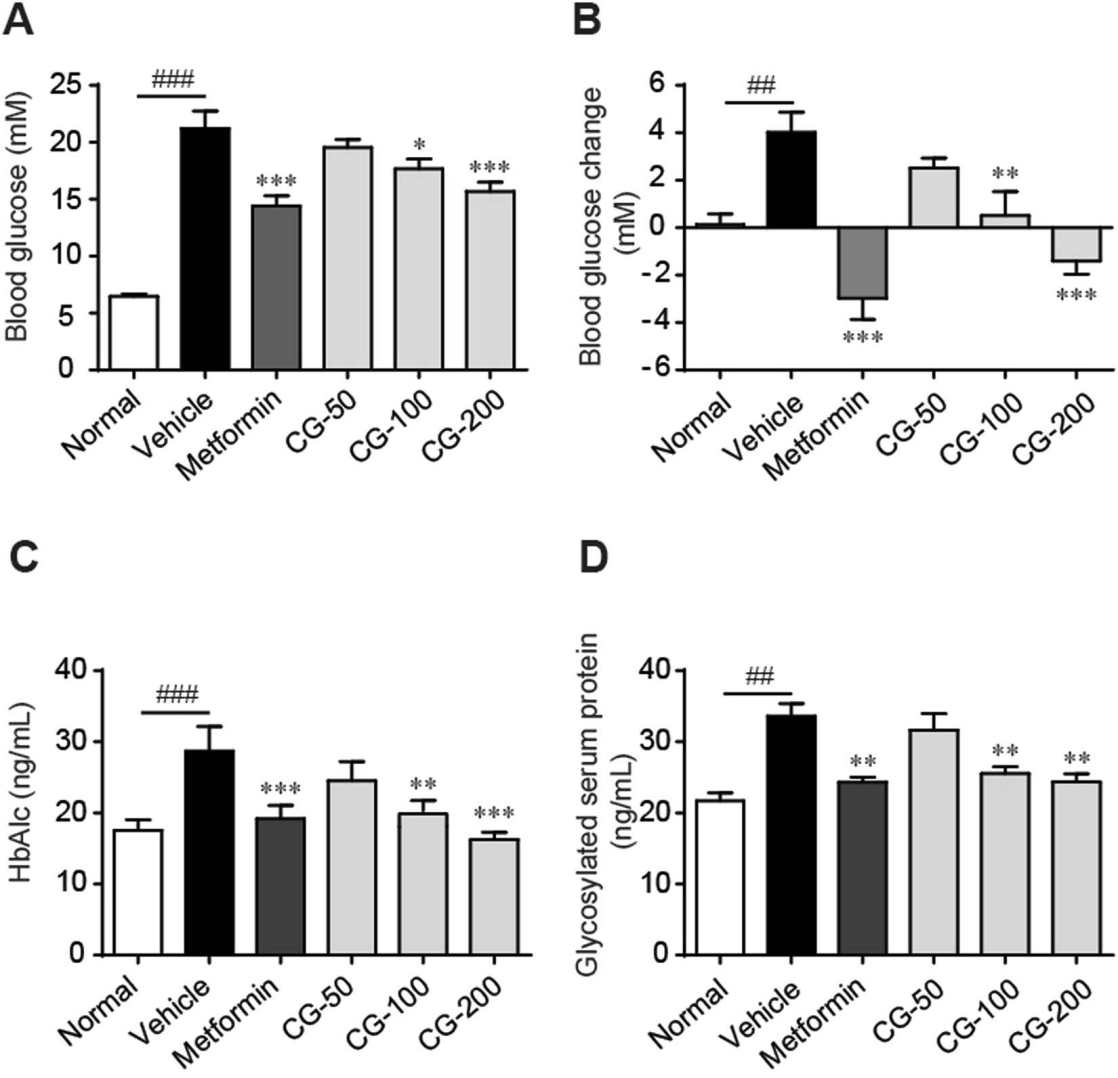
CG alleviated hyperglycemia in ob/ob mice. (**A**) Fasting blood glucose. (**B**) Blood glucose changes during the 4-week period. (**C**) Serum HbAlc level. (**D**) Glycosylated serum protein (GSP) level. (**E**) Fasting serum insulin. (**F**) Homeostasis model assessment-estimated IR (HOMA-IR) index. Six animals in each group.^##^*p* < 0.01, ^###^*p* < 0.001, normal group vs vehicle control group; *p < 0.05, **p < 0.01, ***p < 0.001, medication groups vs vehicle control group.

During the 4-week experimental period, the blood glucose level of ob/ob mice was significantly increased and resulted in hyperglycemia (Fig. 3A and B). The serum levels of glycosylated hemoglobin (HbAlc) and glycosylated serum protein (GSP) were also increased in ob/ob mice as compared to the lean control mice (Fig. 3C and D). Administration of CG significantly and dose-dependently decreased the fasting blood glucose, prevented blood glucose increase, and reduced serum levels of HbAlc and GSP (Fig. 3A–D). The efficacy of CG was comparable to that of metformin. The serum levels of alanine aminotransferase (ALT), aspartate aminotransferase (AST) and lactate dehydrogenase, that are associated with liver injury, were not significantly changed, indicating that anti-hyperglycemic effects of CG were not due to toxic response (Fig. S2).

### CG alleviated insulin resistance

**Figure 4:**
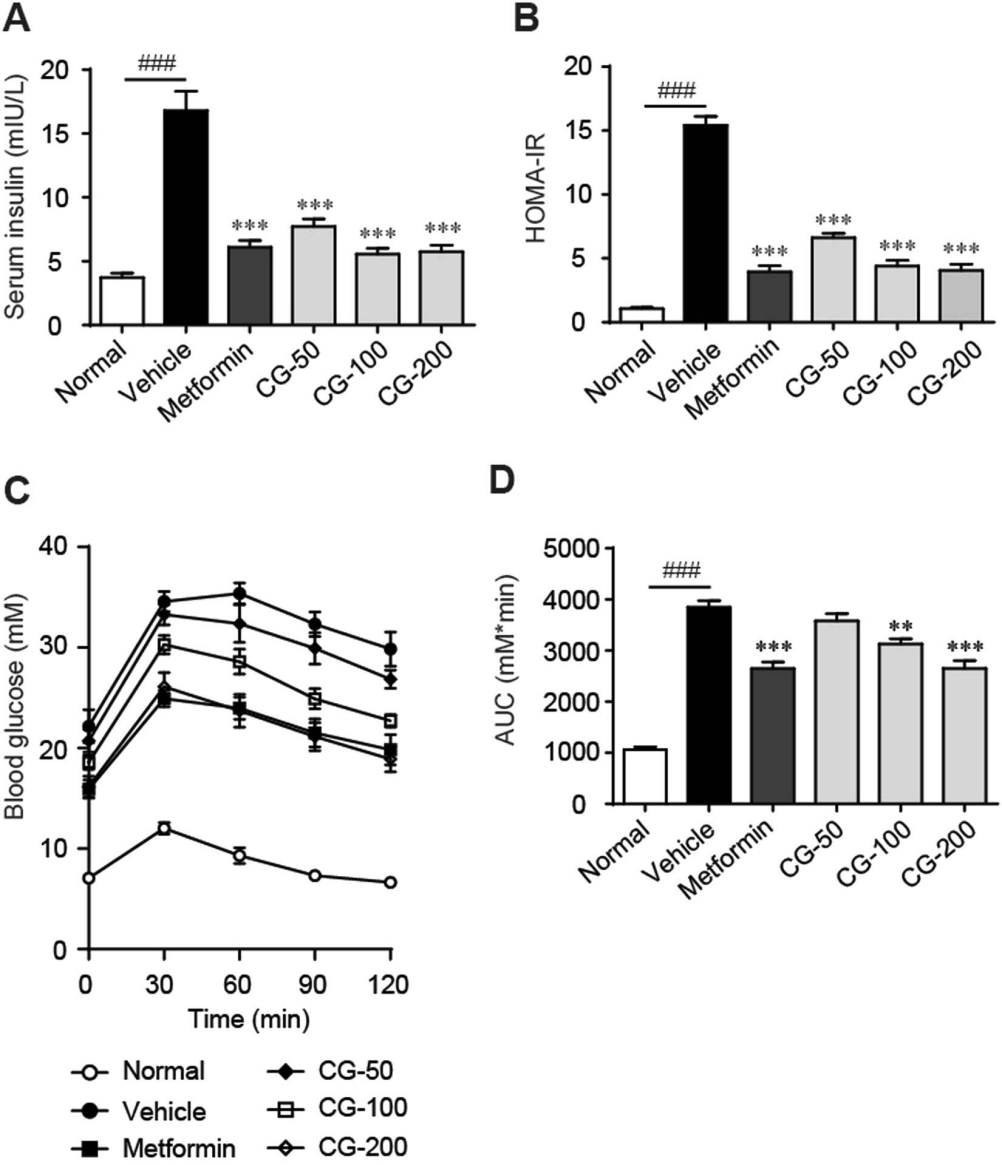
CG improved oral glucose tolerance (OGTT). (**A**) OGTT curve. (**B**) Area under the curve (AUC_glucose_). Six animals in each group. ^###^*p* < 0.001, normal group vs vehicle control group; **p < 0.01, ***p < 0.001, medication groups vs vehicle control group.

Compared with the lean control mice, diabetic ob/ob mice exhibited remarkable insulin resistance, as manifested by significant higher fasting serum insulin level (Fig. 4A) and homeostasis model assessment-estimated IR (HOMA-IR) index (Fig. 4B), and deteriorated oral glucose tolerance (Fig. 4C and D). Treatment with CG significant decreased fasting serum insulin level and HOMA-IR (Fig. 4A and B), and dramatically improved oral glucose tolerance (Fig. 4C and D). These results indicated that CG might alleviate T2DM through improving insulin sensitivity.

### CG ameliorated hyperlipidemia

**Figure 5:**
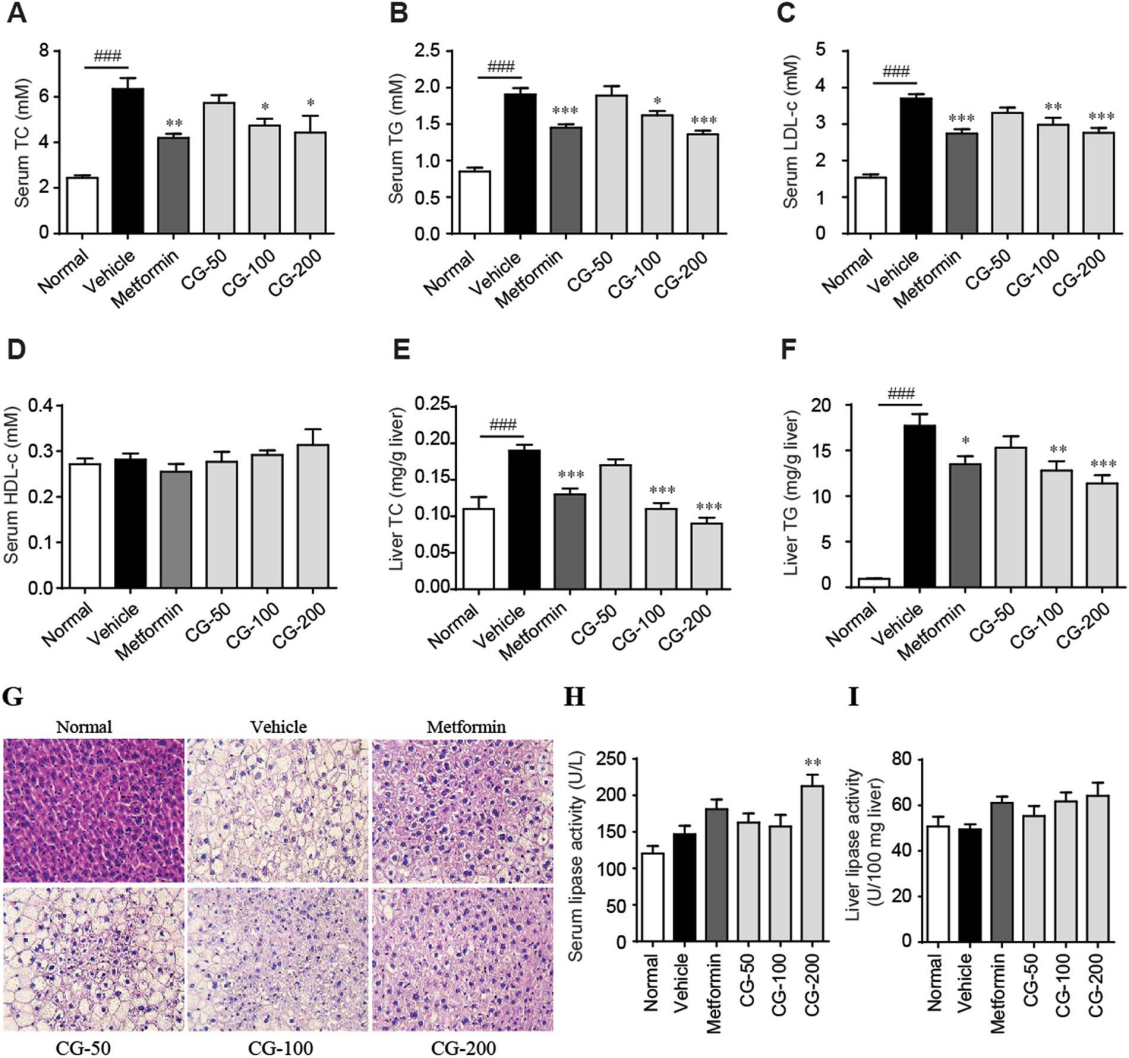
CG reduced hyperlipidemia in ob/ob mice. (**A**) Serum total cholesterol (TC). (**B**) Serum triglycerides (TG). (**C**) Serum low-density lipoprotein cholesterol (LDL-c). (**D**) Serum HDL-c. (**E**) Liver TC. (**F**) Liver TG. (**G**) Hematoxylin and eosin (H&E) staining of liver. (**H**) Serum lipase activity. (I) Liver lipase activity. Six animals in each group. ^##^*p* < 0.01, ^###^*p* < 0.001, normal group vs vehicle control group; *p < 0.05, **p < 0.01, ***p < 0.001, medication groups vs vehicle control group.

Diabetic patients are usually associated with hyperlipidemia and liver steatosis. As determined by biochemical analysis of the serum and liver parameters, diabetic ob/ob mice showed significantly increased serum levels of triglyceride (TG), total cholesterol (TC), and low-density lipoprotein cholesterol (LDL-c) (Fig. 5A–C) though the high-density lipoprotein cholesterol (HDL-c) was not significantly changed (Fig. 5D). Their livers also showed obvious steatosis as the liver levels of TC and TG were largely increased and the morphology of liver by H&E staining exhibited obvious lipid accumulation and fatty degeneration (ballooning) of hepatocytes (Fig. 5E–G). Treatment with CG (100 and 200 mg/kg) substantially reduced the elevated levels of TC, TG and LDL-c in both the bloodstream and liver (Fig. 5A–F) and ameliorated liver steatosis (Fig. 5G).

Lipase is a key enzyme for the mobilization and hydrolysis of triglycerides. The dramatic decrease of serum and liver levels of TG by CG promoted us to examine the effect of CG on the activity of serum and liver lipase. As shown in Fig. 5H and I, CG increased the activity of both serum and liver lipases and significantly increased serum lipase activity at the dose of 200 mg/kg, which suggested that CG may alleviate hyperlipidemia through enhancing the activity of serum and liver lipase.

### CG reduced inflammation

**Figure 6:**
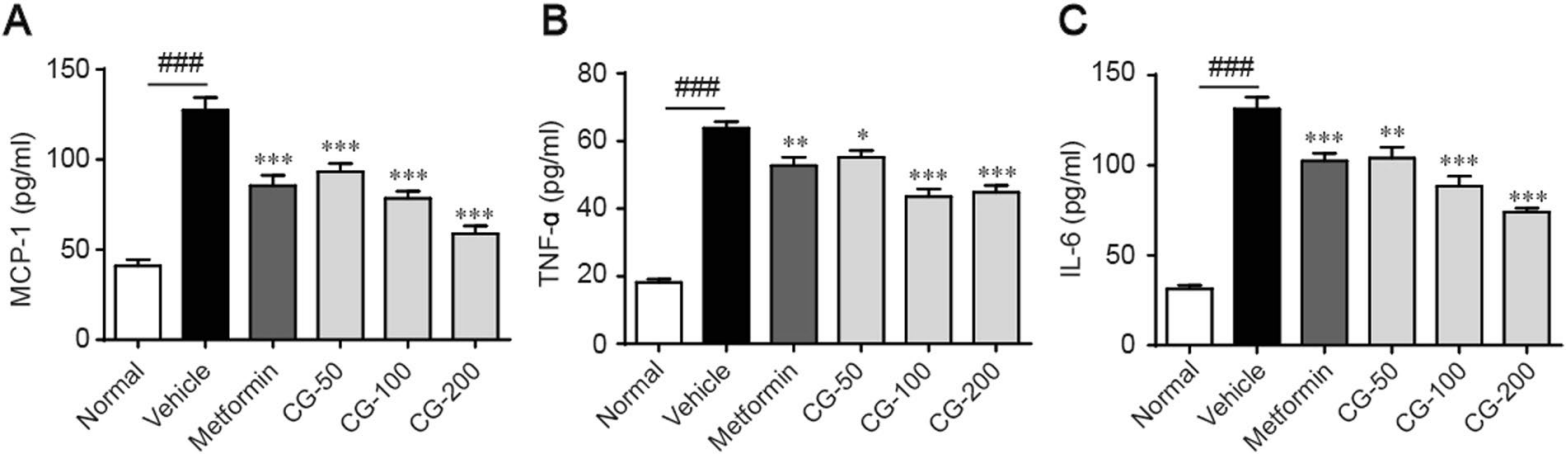
CG markedly ameliorates inflammation. Serum levels of monocyte chemotactic protein-1 (MCP-1) (**A**), tumor necrosis factor ɑ (TNF-ɑ) (**B**) andinterleukin-6 (IL-6) (**C**) are quantified by specific kits. Six animals in each group. ^##^*p* < 0.01, ^###^*p* < 0.001, normal group vs vehicle control group; **p < 0.01, ***p < 0.001, test groups vs vehicle control group.

Chronic inflammation is a common character of diabetes, which is a key factor for the development of insulin resistance^1,13^. As shown in Fig. 6, the serum levels of monocyte chemotactic protein-1 (MCP-1), tumor necrosis factor alpha (TNFα) and interleukelin-6 (IL-6) were significantly higher in ob/ob diabetic mice as compared to the normal control, revealing an obvious inflammatory condition. Treatment with CG largely suppressed the elevated levels of these pro-inflammatory factors, suggested that CG is effective to reduce diabetes-associated inflammation.

### CG stimulated liver glycolysis and promoted glycogen production

**Figure 7:**
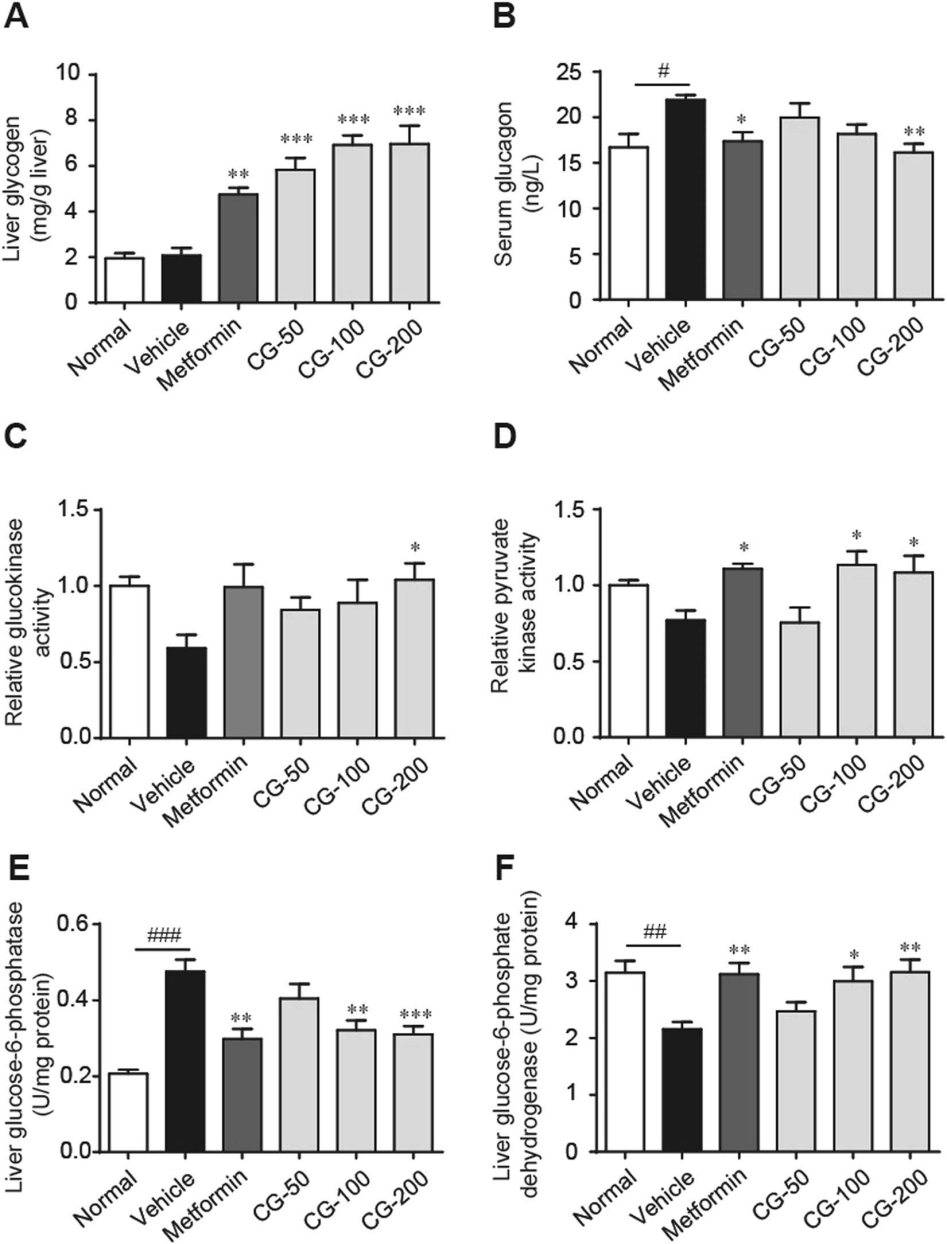
CG decreased serum glycagen and increased liver glycogen production. (**A**) Liver glycogen level. (**B**) Serum glucagon level. (**C**) Liver glucokinase activity. (**D**) Liver pyruvate kinase activity. (**E**) Liver glucose-6-phosphatase activity. (**F**) Liver glucose-6-phosphate dehydrogenase activity. Six animals in each group. ^#^*p* < 0.05, ^##^*p* < 0.01, normal group vs vehicle control group; *p < 0.05, **p < 0.01, ***p < 0.001, medication groups vs vehicle control group.

The disordered glucose metabolism in the liver largely contributes to the abnormal blood glucose level. The pyruvate kinase, glucokinase, glucose-6-phosphatase and glucose-6-phosphate dehydrogenase are key enzymes involved in glucose metabolism, in which glucokinase, glucose-6-phosphate dehydrogenase and pyruvate kinase participate in glucolysis while glucose-6-phosphatase promotes live glucogen degradation and gluconeogenesis. The hepatic activity of pyruvate kinase, glucose-6-phosphate dehydrogenase and glucokinase activities was remarkably decreased while glucose-6-phosphatase activity was increased in ob/ob mice as compared with the lean animals (Fig. 7C–F), though the glycogen content showed no significant difference (Fig. 7A). Treatment with CG dose-dependently increased the hepatic content of glycogen and the hepatic activity of pyruvate kinase, glucose-6-phosphate dehydrogenase and glucokinase, and decreased the activity of glucose-6-phosphatase (Fig. 7), suggesting that CG stimulated the liver glycolysis and inhibited gluconeogenesis

Hypersecretion of glucagon, a peptide hormone secreted from pancreatic α cells in response to hypoglycemia^14^, is an important pathogenic factor for the development of T2DM^15^. The diabetic ob/ob mice exhibited significant increase in serum glucagon level and treatment with CG dose-dependently reversed the serum level of glucagon (Fig. 7B).

## Discussion

*L. glutinosa* is a traditional herbal medicine that is broadly used for the treatment of diarrhea, dysentery, poultice sprains and bruises^6^. In the present study, we demonstrated for the first time that the CG displayed adequate anti-diabetic effect in genetic T2DM ob/ob mice. Oral administration of CG for 4 weeks dose-dependently decreased the fasting blood glucose, and prevented blood glucose increase. The serum levels of HbAlc and GSP were also significantly reduced. The efficiency of CG (200 mg/kg) in decreasing serum levels of fasting glucose, HbAlc and GSP were comparable to that of metformin (200 mg/kg). These results suggested that CG is an effective anti-hyperglycemic agent.

Insulin resistance is a central symptom of T2DM^16^. Administration of CG substantially reduced insulin resistance in ob/ob mice. First, the hyperinsulinemia was substantially alleviated, as manifested by the dramatic decrease in the serum level of fasting insulin. Accordingly, the insulin resistance indicator HOMA-IR was dramatically declined. Moreover, CG treatment significantly improved the oral glucose tolerance and the area under the OGTT curve was decreased by CG in a dose-dependent manner. These results demonstrated that CG is effective to ameliorate insulin resistance of diabetic animals. Interestingly, CG did not decrease the average food intake, suggesting that the alleviating effect of CG on diabetes was not due to food restriction.

Obesity, hyperlipidemia, and inflammation are commonly associated with T2DM and play a key role in the development of insulin resistance. Administration of CG effectively reduced obesity in ob/ob mice, as manifested by the significant decrease in body and fat (subcutaneous fat and epidy-dimal fat) weights. Treatment with CG (100 and 200 mg/kg) also alleviated hyperlipidemia in ob/ob mice. The increased levels of serum TC, TG and LDL-c by high fat-diet were substantially decreased after CG administration. The liver steatosis was also effectively ameliorated, as manifested by the reduced hepatic TC, TG and histological steatosis. Similarly, the inflammation in ob/ob mice was largely alleviated. The serum levels of MCP-1, TNFα and IL-6 were significantly decreased by CG. These data suggested that CG might ameliorate insulin resistance through alleviating obesity, hyperlipidemia and inflammation.

Liver is a main spot for glucose metabolism. After feeding, insulin stimulates liver to take more glucose from the blood and synthesize glycogen, thus reducing the postprandial hyperglycemia. At the same time, glycolysis is also increased to degrade the excessive glucose. Under diabetic condition, glycogen synthesis and glycolysis are largely inhibited due to insulin resistance. Our results showed that CG significantly enhanced the activity of liver glucokinase, a key enzyme in glycogen synthesis^17^, and increased the content of hepatic glycogen. The activity of liver pyruvate kinase was also increased by CG. At the same time, CG significantly decreased the serum level of glucagon, a main catabolic hormone that raises the concentration of glucose and fat in the blood stream^18,19^. These results suggest that CG may reduce blood glucose levels by stimulating hepatic glycogen synthesis, increasing glycolysis and decreasing serum glucagon.

Metformin is the first-line medication for oral diabetes treatment^20^. It specifically reduces hepatic gluconeogenesis without increasing insulin secretion, inducing weight gain or posing a risk of hypoglycaemia^21^. Our data showed that oral administration of CG exhibited similar effects with metformin in decreasing blood glucose, alleviating insulin resistance, improving dyslipidemia and preventing inflammation. Overall,the efficiency of CG (200 mg/kg) was comparable to metformin (200 mg/kg). There were also some difference between the anti-diabetic effects of CG and metformin. First, treatment with metformin significantly decreased body weight and declined average food intake. In contrast, oral administration of CG at 200 mg/kg also prevented bodyweight gain but showed less influence on food intake. Second, although the blood glucose decrease by CG (200 mg/kg) was slightly smaller than by metformin (200 mg/kg), the serum level of HbA1c was more pronouncedly declined by CG. These results suggested that CG was more effective to maintain blood glucose level during a long period. Third, CG displayed a similar impact on blood lipids with metformin but more efficiently decreased hepatic lipids than metformin. Forth, CG was more potent in alleviating inflammation as the blood levels of inflammatory factors such as MCP-1, TNFα and IL-6 were more pronouncedly decreased by CG than metformin. At last, CG and metformin inhibited activity of glucose-6-phosphatase and increased the activity of glucokinase, glucose-6-phosphate dehydrogenase and pyruvate kinase in a same degree, revealing similar effects on hepatic glycolysis and glucogenesis. Although both CG and metformin significantly increased the liver glycogen, the liver content of glycogen in CG-treated mice was much higher than metformin-treated animals, suggesting that CG was more efficient to promote glycogen accumulation and thus decrease blood glucose. All together, our results provided solid evidence proving that CG is an effective agent for the treatment of diabetes. UPLC-QTOF-MS analysis showed that CG was rich in alkaloid, especially aporphine type alkaloids. Laurolitsine was the characteristic compound of *L. glutinosa* and was the most abundant one. Apart from this compound, seven main alkaloids were also identified. Aporphine alkaloids have been investigated for their anti-hyperglycemic and anti-hyperlipidemia effects^22,23^. Laurolitsine and boldine were found to have the ability of lowering glucose uptake by isolated intestinal brush-border membrane vesiclesor basolateral membrane vesicles and glucose absorption during *in situ* intestinal perfusion^24^. Additionally, Intravenous injection of boldine decreased the plasma glucose levels in a dose-dependent manner in both normal and diabetic rats^25^. Other researches also confirmed the anti-hyperglycemic and anti-hyperlipidemia of boldine^26,27^. These studies verified that aporphine alkaloids have the ability in anti-hyperglycemic and anti-hyperlipidemia. The potential mechanism in charging of anti-hyperglycemic may attribute to the stimulation of insulin release and the increase of glucose utilization, but still need detailed investigations.

Laurolitsine was the richest compound in CG and its structure was very similar to boldine with the loss of the *N*-methyl. Therefore, laurolitsine could be deemed as the active ingredient of CG. Although, there has been a report regarding the anti-hyperglycemic activity of laurolitsine, systematic investigations should be carried out both on its effects and mechanism as well as its toxicity. In the near future, we will deeply study its beneficial effects on diabetes and mechanisms after getting enough amounts.

## Methods

### Plant materials

The barks of *L. glutinosa* were collected in Tong Gu Ling, Wenchang city, Hainan Province in May, 2017 and were identified by Professor Niankai Zeng. A voucher specimen (LG-7-17) has been deposited in Hainan Medical University.

### Extraction and isolation of CG

The barks of *L. glutinosa* was dried and powdered and then extracted by ethanol under reflux. The ethanol extract was concentrated under reduced pressure to give a residue. The residue was dissolved in water, and then its pH value was adjusted to 2 by adding 1% H_2_SO_4_. The acidic solution was partitioned by chloroform to remove the lipid-soluble compounds. Then the pH value of the residue was adjusted to 10 by adding ammonia. Further, ethyl acetate was used to extract the basic solution, after concentration, to give the alkaloid-rich extract of *Litsea glutinosa* barks, which we called CG in this work.

### UPLC-QTOF-MS analysis

The LC-MS analysis was performed on an Agilent 1290 Series UHPLC system coupled to an Agilent 6540 TOF/MS instrument with a Dual AJS ESI source. Chromatographic separation was achieved using an ACQUITY UPLC BEN (2.1 mm × 100 mm, 1.7 μm particle) analytical column operated at 60 °C. The mobile phase consisted of 0.1% formic acid (v/v) in water (A) and acetonitrile (B) at a flow of 0.30 mL·min^−1^. The gradient started from 5% B to 13% B in 3 min, followed by 13% B to 100% B in 20 min, and held for 2 min, then returned the initial gradient composition and allowed to equilibrate for 4 min. Mass spectrometric analysis were collected in positive mode with full scan mode from 50 to 1000* m*/*z*. The reference masses (121.0509* m*/*z* for C_5_H_4_N_4_, and 922.0098 for C_18_H_18_O_6_N_3_P_3_F_24_) were continuously measured during the analysis to allow constant mass correction. The electrospray source was operated at 350 °C with an ionization voltage of 3500 V; the nebulizer gas pressure and the drying gas flow rate were set at 40 psig and 12 L/min, the fragmentor was 130 V and the skimmer voltage was 65 V, respectively. Product ions were performed in target MS/MS mode from 50 to 800* m*/*z*, and the collision energy was 10, 20 and 30 ev.

### Animals and experimental design

All the animal experiments were performed in accordance with the National Institutes of Health regulations for the care and use of animals in research. The experimental protocols were approved by the Medical Ethics Committee of Peking Union Medical College (No. YZS201703010).

To evaluate the anti-diabetic activity of CG, thirty male ob/ob mice, 12-week old, weighing 50–55 g, and six male C56BL6 mice, 12-week old, weighing 28–32 g, were purchased from Vital River Laboratory Animal Technology Co., Ltd. (Beijing, China). The animals were kept in a humidity-controlled room on a 12-h light-dark cycle with food and water available *ad libitum* for one week, and then divided randomly into six groups with six animals in each group. The normal and vehicle control groups were given equal volume of solvent (0.5% sodium carboxymethyl cellulose (CMC-Na)). The positive control group was treated with 200 mg/kg of metformin while the CG groups were administrated with 50, 100 or 200 mg/kg CG via gavage. The body weight and food intake were measured once a week. At the end of the 4-week period, after the animals were fasted overnight, each animal was weighed, and blood samples were collected. Animals were then euthanized, and the weights of the liver, subcutaneous fat and epidy-dimal fat were determined. A piece of liver was taken from each animal and fixed for histological analysis.

### Oral glucose tolerance test

On the 24th day after CG treatment, oral glucose tolerance test (OGTT) was performed on all animals. OGTT using 2 g/kg of glucose was conducted as described^26^. Blood samples were collected from tail vein for glucose measurement at 0, 30, 60, 90 and 120 min after glucose administration (po). The blood glucose levels were determined by a glucose meter (Roche, ACCU-CHEK Active).

### Biochemical analysis

All serum and hepatic biochemical parameters except serum insulin were measured by respective kits (Jian Cheng Biotechnology Company, Nanjing, China) according to the manufacturer’s instruction. Serum insulin levels were determined byradio immunoassay kit (Beijing North Institute of Biological Technology, Beijing, China) according to the manufacturer’s instruction. The activities of liver glucokinase (Jian Cheng Biotechnology Company), glucose 6-phosphatase and glucose 6-phosphate dehydrogenase (Biovision, USA), and serum aspartate aminotransferase (AST) and alanine aminotransferase (ALT) (Jian Cheng Biotechnology Company) were assayed by respective kits according to the manufacturer’s instruction.

### Histological analysis

The liver of each animal were fixed in 4% buffered neutral formalin, embedded in paraffin and cut at 4 μm. The sections were stained with hematoxylin and eosin (H&E), and their morphological changes were evaluated. The steatotic rate of liver was determined by counting the number of steatotic cells vs. the total cell number in each visual field. At least 15 slides of each group were analyzed.

### Statistical analysis

Data are expressed as mean ± S.E.M. One-way ANOVA was used to determine significant differences among groups, after which the modified Student’s t-test with the Bonferroni correction was used for comparison between individual groups. All statistical analyses were performed with SPSS 17.0 software (SPSS Inc., Chicago, IL, USA). *p* < 0.05 was considered statistically significant^28–30^.

## Electronic supplementary material

Supplementary information

## Data Availability

All data generated or analyzed during this study are included in this published article and its Supplementary Information files.

## References

[1] Bhowmick R (2014). *In vivo* analgesic, antipyretic, and anti-inflammatory potential in Swiss albino mice and *in vitro* thrombolytic activity of hydroalcoholic extract from Litsea glutinosa leaves. Biol Res.

[2] Mandal SC, Kumar CK, Majumder A, Majumder R, Maity BC (2010). Antibacterial activity of Litsea glutinosa bark. Fitoterapia.

[3] O’Brien SH, Koch T, Vesely SK, Schwarz EB (2017). Hormonal Contraception and Risk of Thromboembolism in Women With Diabetes. Diabetes Care.

[4] Wu C (2014). Cordycepin activates AMP-activated protein kinase (AMPK) via interaction with the gamma1 subunit. J Cell Mol Med.

[5] Patti ME (2003). Coordinated reduction of genes of oxidative metabolism in humans with insulin resistance and diabetes: Potential role of PGC1 and NRF1. Proc Natl Acad Sci USA.

[6] Cusi K (2000). Insulin resistance differentially affects the PI 3-kinase- and MAP kinase-mediated signaling in human muscle. J Clin Invest.

[7] Eriksson J (1989). Early metabolic defects in persons at increased risk for non-insulin-dependent diabetes mellitus. N Engl J Med.

[8] Das D, Maiti S, Maiti TK, Islam SS (2013). A new arabinoxylan from green leaves of Litsea glutinosa (Lauraeae): structural and biological studies. Carbohydr Polym.

[9] Agrawal N (2013). Butanolides from methanolic extract of Litsea glutinosa. Chem Biodivers.

[10] Herath HM, Kumar NS, Wimalasiri KM (1990). Structural studies of an arabinoxylan isolated from Litsea glutinosa (Lauraceae). Carbohydr Res.

[11] Wang YS, Huang R, Lu H, Li FY, Yang JH (2010). A new 2’-oxygenated flavone glycoside from Litsea glutinosa (Lour.) C. B. Rob. Biosci Biotechnol Biochem.

[12] Wu Y (2017). New lignan glycosides from the root barks of Litsea glutinosa. Phytochemistry Letters.

[13] Mandal S, Kumar C, Majumder A, Majumder R, Maity BC (2000). Antibacterial activity of Litsea glutinosa bark. Fitoterapia.

[14] Chang SK (2017). Stromal cell cadherin-11 regulates adipose tissue inflammation and diabetes. J Clin Invest.

[15] Chen Q (2017). Berberine Ameliorates Diabetes-Associated Cognitive Decline through Modulation of Aberrant Inflammation Response and Insulin Signaling Pathway in DM Rats. Front Pharmacol.

[16] Wewer Albrechtsen NJ (2017). Circulating glucagon 1-61 regulates blood glucose by increasing insulin secretion and hepatic glucose production. Cell Rep.

[17] Unger RH, Cherrington AD (2012). Glucagonocentric restructuring of diabetes: a pathophysiologic and therapeutic makeover. J Clin Invest.

[18] Jiang B (2016). Protective effects of marein on high glucose-induced glucose metabolic disorder in HepG2 cells. Phytomedicine.

[19] Pari L, Saravanan R (2004). Antidiabetic effect of diasulin, a herbal drug, on blood glucose, plasma insulin and hepatic enzymes of glucose metabolism in hyperglycaemic rats. Diabetes Obes Metab.

[20] Gastaldelli A, Gaggini M, DeFronzo R (2017). Glucose kinetics: an update and novel insights into its regulation by glucagon and GLP-1. Curr Opin Clin Nutr Metab Care.

[21] Okamoto H (2017). Glucagon receptor inhibition normalizes blood glucose in severe insulin-resistant mice. Proc Natl Acad Sci USA.

[22] UK Prospective Diabetes Study (UKPDS) Group (1998). Effect of intensive blood-glucose control with metformin on complications in overweight patients with type 2 diabetes (UKPDS 34). Lancet.

[23] Madiraju AK (2014). Metformin suppresses gluconeogenesis by inhibiting mitochondrial glycerophosphate dehydrogenase. Nature.

[24] Nguyen K (2012). Nuciferine stimulates insulin secretion from beta cells-an *in vitro* comparison with glibenclamide. J Ethnopharmacol.

[25] Yu B, Cook C, Santanam N (2009). The aporphine alkaloid boldine induces adiponectin expression and regulation in 3T3-L1 cells. J Med Food.

[26] Lin C (2006). Inhibition of intestinal glucose uptake by aporphines and secoaporphines. Life Sci.

[27] Chi T, Lee S, Su M (2006). Antihyperglycemic effect of aporphines and their derivatives in normal and diabetic rats. Planta Med.

[28] Lau Y (2013). Boldine protects endothelial function in hyperglycemia-induced oxidative stress through an antioxidant mechanism. Biochem Pharmacol.

[29] Lau Y, Ling W, Murugan D, Mustafa M (2015). Boldine Ameliorates Vascular Oxidative Stress and Endothelial Dysfunction: Therapeutic Implication for Hypertension and Diabetes. J Cardiovasc Pharmacol.

[30] Wu C (2014). The caffeoylquinic acid-rich Pandanus tectorius fruit extract increases insulin sensitivity and regulates hepatic glucose and lipid metabolism in diabetic db/db mice. J Nutr Biochem.

