# Supplementary material for: Anti-hyperglycemic and anti-hyperlipidemia effects of the alkaloid-rich extract from barks of Litsea glutinosa in ob/ob mice
Source: Sci Rep. 2018 Aug 23;8:12646. doi: 10.1038/s41598-018-30823-w (PMC6107583; doi:10.1038/s41598-018-30823-w)
Supplement: Supplementary file 1 — Supplementary information [file 41598_2018_30823_MOESM1_ESM.pdf]

Supplementary information

Anti-hyperglycemic and anti-hyperlipidemia effects of the alkaloid-rich extract from barks of Litsea glutinosa in ob/ob mice

Xiaopo Zhang[1], Yan Jin[1], Younan Wu[1], Caiyun Zhang[1], Dejun Jin[1], Qingxia Zheng*[2], and Youbin Li*[1]

*Correspondence and requests for materials should be addressed to Q. Z. (zhengqingxia916@126.com) and Y. L. (liyoubinli@sohu.com)

This PDF includes:
Supplementary Table S1
Supplementary Figures S1-S2

No.	Compounds	Retention time (min)	Ion peaks
1	Laurelliptine	3.642	314.1392 $[M+H]^+$
2	6-Isoquinolinol	5.449	286.1436 $[M+H]^+$
3	Laurolitsine	5.681	314.1390 $[M+H]^+$
4	Isoboldine	10.219	328.1549 $[M+H]^+$
5	*N*-methyl laurolitsine	8.719	330.1718 $[M+H]^+$
6	Laurolitsine	12.167	300.1605 $[M+H]^+$
7	Boldine	14.733	328.1541 $[M+H]^+$
8	Litseglutine A	15.302	312.1223 $[M+H]^+$

Supplementary Table S1 | The name, retention time, ion peaks of the compounds identified in CG.

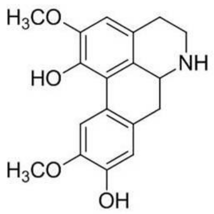

1

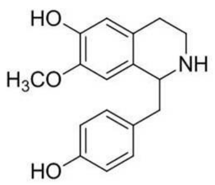

2

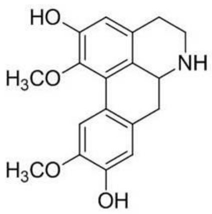

3

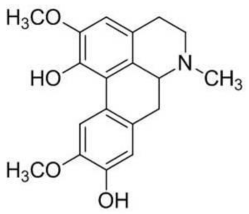

4

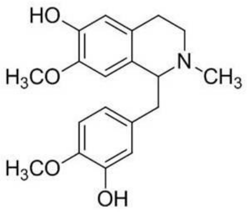

5

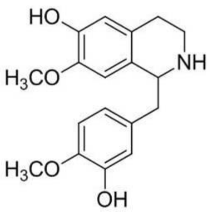

6

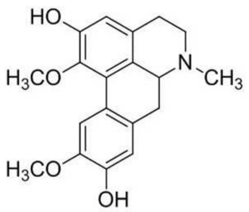

7

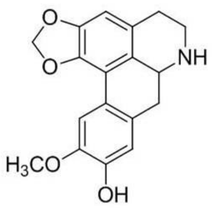

8

Supplementary Figure S1 | Chemical structures of the compounds identified in CG.

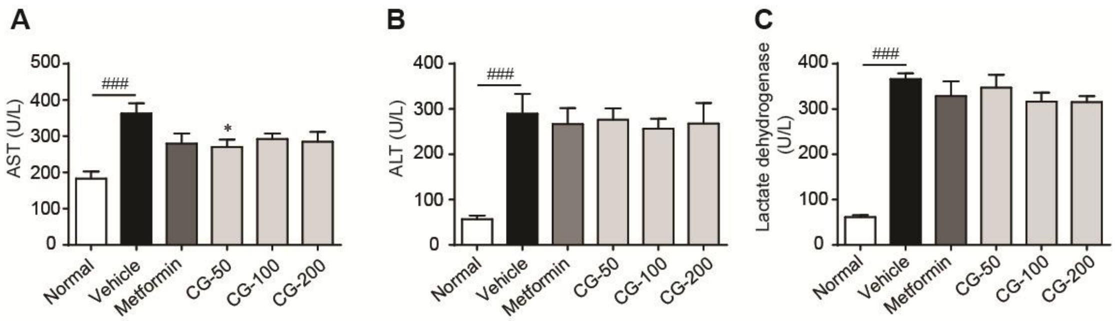

Supplementary Figure S2 | CG showed no toxicity on liver (n=6). (A) Serum aspartate transaminase (AST). (B) Serum alanine transaminase (ALT).Six animals in each group. ###p<0.001, normal group vs vehicle control group.
